# The Importance of a Multidisciplinary Team Approach in a Rare Case of Antenatally Diagnosed Tonne-Kalscheuer Syndrome

**DOI:** 10.7759/cureus.101077

**Published:** 2026-01-08

**Authors:** Brittany Woody, Malek Asfar, Alexander Berra, Divya Chilukuri, Ruth Villarosa, Chandini Madeswaran, Cameron Friedman, Riya Kalra, Deepak Kumar, Rocio Moran, Mariam Youssef

**Affiliations:** 1 Psychiatry, Alabama College of Osteopathic Medicine, Dothan, USA; 2 Pathology and Laboratory Medicine, Case Western Reserve University/MetroHealth Medical Center, Cleveland, USA; 3 Obstetrics and Gynecology, MetroHealth Medical Center, Cleveland, USA; 4 Palliative Care, MetroHealth Medical Center, Cleveland, USA; 5 Pathology, MetroHealth Medical Center, Cleveland, USA; 6 Genetics, MetroHealth Medical Center, Cleveland, USA; 7 Neonatal-Perinatal Medicine, MetroHealth Medical Center, Cleveland, USA

**Keywords:** holistic care for patients, multidisciplinary care team, palliative management, tokas, tonne-kalscheuer syndrome

## Abstract

Tonne-Kalscheuer syndrome (TOKAS) is a rare X-linked neurodevelopmental disorder with very few reported cases. We describe the case of a male neonate born at 27 weeks’ gestation to a 41-year-old mother, with a diagnosis of TOKAS established antenatally through whole-exome sequencing after ultrasound revealed nuchal translucency, hydrops fetalis, structural cardiac abnormalities, and severe fetal growth restriction. The infant survived for nearly two hours after birth and was cared for and held by his parents and extended family. This case highlights the essential role of multidisciplinary collaboration, including maternal-fetal medicine, genetics, neonatology, pathology, and palliative care, in providing coordinated, compassionate, and family-centered care in the setting of a life-limiting fetal diagnosis. It also contributes to the limited literature on the prenatal presentation and perinatal course of TOKAS.

## Introduction

Tonne et al. first described a novel X-linked intellectual disability syndrome in 2015, identifying a missense mutation in the *RLIM* gene located at Xq13.2 as the underlying cause [1]. Subsequent reports by Frints et al. [2] confirmed that additional pathogenic variants in *RLIM* were associated with X-linked neurodevelopmental disorders. As recognition of this entity expanded, a broader constellation of clinical features emerged, reflecting the phenotypic variability associated with *RLIM* mutations. This multiple congenital anomaly syndrome is now known as Tonne-Kalscheuer syndrome (TOKAS) (OMIM #300978) [3]. To date, only 18 antenatally diagnosed cases have been reported.

In the original family described by Tonne et al. [1], a Tyr356Cys missense mutation was identified in four affected individuals [1]. Since then, nine additional missense variants in *RLIM* have been documented across the literature with variable severity [4]. Among these, Arg611Cys is the most frequently reported and is considered one of the most deleterious variants.

The clinical phenotype of TOKAS is diverse. Affected children may exhibit neurodevelopmental impairments, including motor and speech delays, autism spectrum features, and mild to profound intellectual disability [2,4]. Characteristic facial features, short stature, and multisystem congenital anomalies have been described, with some individuals developing seizures, structural brain malformations, or congenital cardiac defects [2,4-6]. Most fetuses with severe presentations do not survive to delivery, often due to congenital diaphragmatic hernia and/or pulmonary hypoplasia, consistent with the high prenatal lethality reported in severe *RLIM* variants [4,7]. Because TOKAS is X-linked, the condition primarily affects males; however, female carriers may exhibit milder manifestations such as short stature, subtle skeletal findings, or hormonal abnormalities, including primary ovarian insufficiency [4]. Despite growing recognition, knowledge about TOKAS remains limited, and ongoing case reporting is essential to further define its genotypic and phenotypic spectrum.

Given the complexity and severity of many presentations, optimal care for affected pregnancies requires a coordinated multidisciplinary team both antenatally and postnatally, including maternal fetal medicine, genetics, neonatology, cardiology, neurology, pathology, and palliative care, to ensure comprehensive counseling, informed decision-making, and high-quality supportive care for families.

## Case presentation

The patient’s mother was a 40-year-old G2P0010 who presented for obstetric care with a confirmed single, living intrauterine pregnancy (IUP) at 8 weeks’ gestation. Her medical history was significant for polycystic ovarian syndrome, class II obesity, depression and anxiety, insulin resistance, obstructive sleep apnea, chronic ethmoidal sinusitis, and migraines. She was an asymptomatic carrier for congenital adrenal hyperplasia due to 21-hydroxylase deficiency. Her surgical history included a Roux-en-Y gastric bypass complicated by stomal stenosis and a suction dilation and curettage for a prior miscarriage. Conception followed five months of ovulation induction using letrozole, luteinizing hormone (Ovidrel®), and follicle-stimulating hormone (Gonal-F®). At the initial eight-week ultrasound, the IUP appeared normal aside from a small subchorionic hemorrhage.

**Figure 1:**
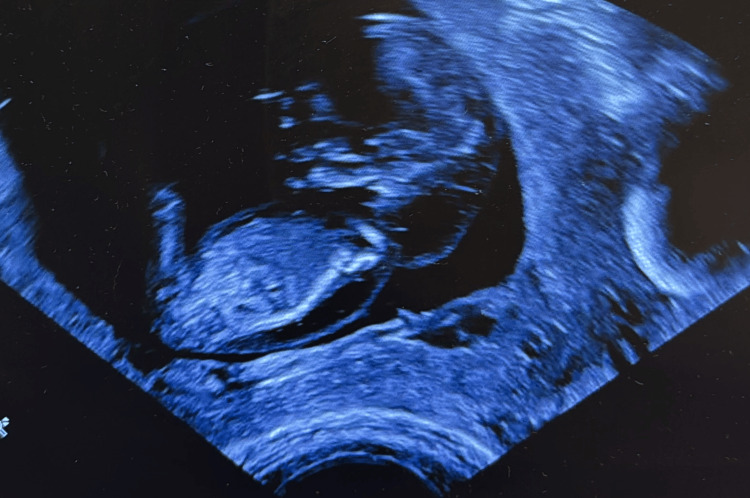
Nuchal translucency on prenatal ultrasound.

**Figure 2:**
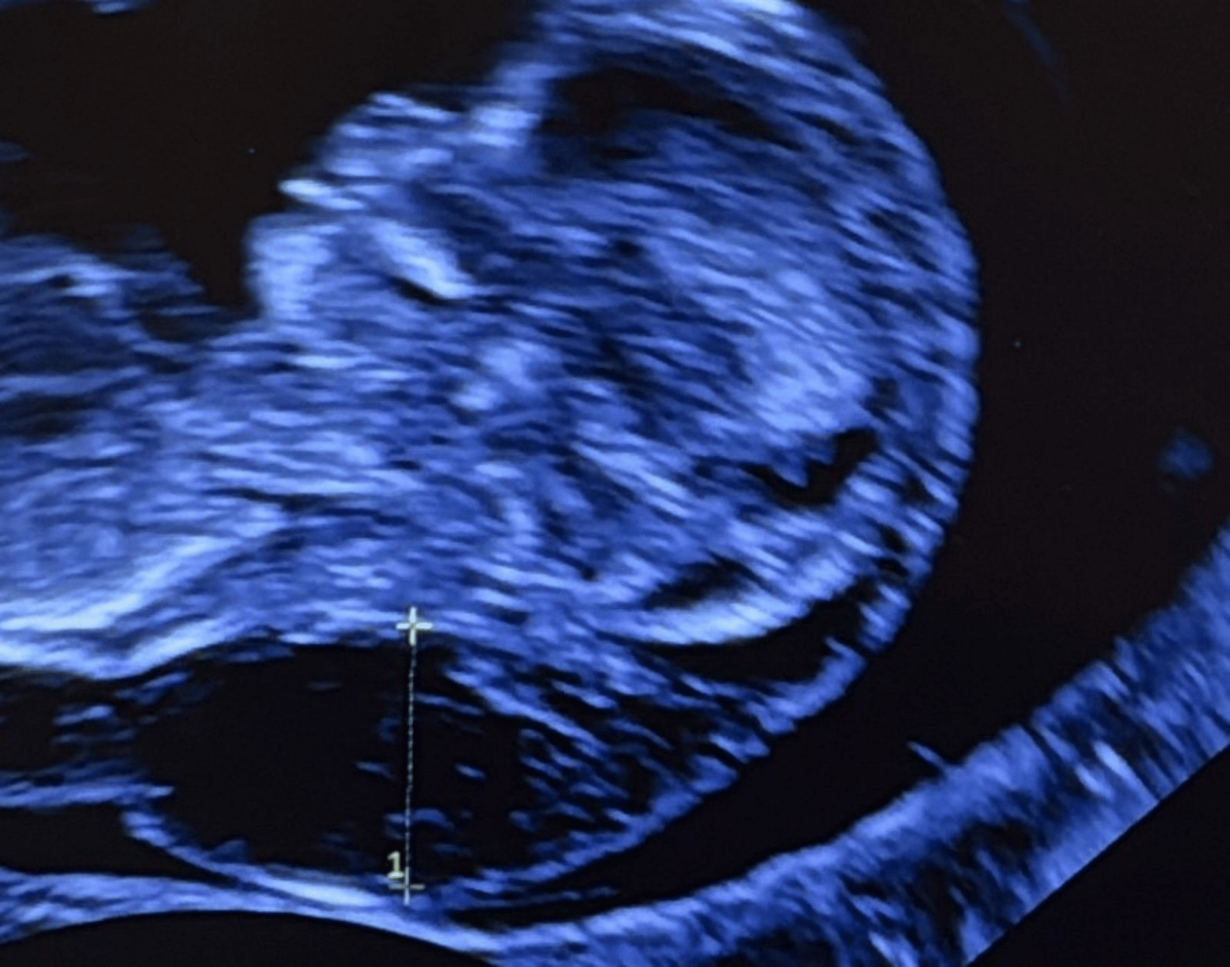
Cystic hygroma on prenatal ultrasound.

Concern for an underlying genetic abnormality arose during the first-trimester nuchal translucency ultrasound performed at 11 weeks 5 days’ gestation (Figure 1). Findings included a markedly enlarged cystic hygroma >95th percentile (Figure 2), reversed a-wave in the ductus venosus, persistent non-visualization of the stomach, a small bladder, and minimal fetal movement. A prenatal genetic counselor reviewed screening and diagnostic options. The mother elected to undergo cell-free fetal DNA screening for expanded aneuploidy and five common microdeletion/duplication syndromes; results were negative. She initially declined invasive testing.

The parents were subsequently offered single-gene cfDNA (cell-free DNA) screening, diagnostic testing, or continued ultrasound surveillance. At 18 weeks 4 days’ gestation, the mother elected amniocentesis. Fluorescence in situ hybridization (FISH) for chromosomes 13, 18, 21, and X/Y chromosomes, single-nucleotide polymorphism (SNP) microarray, and trio whole-exome sequencing (WES) were ordered. Both FISH and SNP microarray results were normal.

While WES results were pending, an anatomy scan at 21 weeks 5 days’ gestation showed poor fetal growth, bilateral cleft lip and palate, suspected congenital heart disease, thickened nuchal fold, and a small cerebellum. A fetal echocardiogram at 24 weeks 1 day gestation demonstrated a mildly hypoplastic aortic valve, a large perimembranous ventricular septal defect (VSD), and a trivial effusion.

Hydrops fetalis was diagnosed at 24 weeks 3 days’ gestation and was found to be progressively worsening on serial ultrasounds. A repeat fetal echocardiogram at 26 weeks 3 days’ gestation revealed worsening hydrops, moderate perimembranous VSD, a trivially hypoplastic mitral valve, possible aortic isthmus narrowing, bilateral superior vena cavae without a bridging vein, and a left superior vena cava draining to the coronary sinus. At 27 weeks 2 days’ gestation, severe fetal growth restriction was noted, with estimated fetal weight below the first percentile.

The neonatology team was consulted at 26 weeks 3 days’ gestation for counseling regarding the anticipated complications related to the extensive congenital anomalies, including cleft lip and palate, significant cardiac defects, and evolving hydrops. At that time, WES remained pending, whereas all other genetic testing had been unremarkable. Counseling included discussion of expected perinatal instability, anticipated need for neonatal intensive care unit (NICU) admission, potential respiratory support, invasive procedures, blood product administration, imaging, and prolonged hospitalization. The parents were reassured that a multidisciplinary team would follow them both antenatally and postnatally to support decision-making and ensure coordinated care for their medically fragile infant.

WES results became available at 27 weeks 4 days’ gestation and revealed a pathogenic maternally inherited *RLIM* missense variant (Arg611Cys), confirming the diagnosis of TOKAS. Given the severe phenotype, progressive hydrops, and the universally poor prognosis reported in the literature, the NICU team revisited goals of care with the parents, together with a palliative care provider. Prior cases of TOKAS have largely resulted in intrauterine demise or neonatal death shortly after birth, with only a single severely affected survivor reported into early childhood. After discussion of the expected futility of aggressive resuscitation, the parents elected a do-not-resuscitate plan.

Because the parents strongly desired a live birth, delivery via cesarean section was recommended to reduce the risk of intrapartum fetal demise. A two-dose course of betamethasone had been initiated at 27 weeks 3 days’ gestation, before confirmation of the genetic diagnosis, although no further steroid administration was planned after transitioning to comfort-focused goals. The perinatal palliative care team provided support, coordinated memory-making activities, and facilitated communication among obstetrics, neonatology, cardiology, genetics, and nursing.

A scheduled cesarean section was performed under epidural anesthesia. At birth, the infant exhibited faint activity, cyanosis, and a heart rate <100 beats/minute. In accordance with the established comfort-care plan, he was dried, wrapped, and immediately placed in the arms of his parents. He lived for 1 hour and 48 minutes, passing peacefully in his mother’s arms.

The organization Now I Lay Me Down to Sleep photographed the family shortly after birth. Additional memory-making activities, including handprints, footprints, and clay impressions, were completed in the hours following his passing, facilitated by the palliative care team and supported using CuddleCot.

A complete autopsy was performed at the parents’ request. Anthropometric measurements were consistent with a 24-25-week gestational-age fetus. All major organ weights, including brain, liver, lungs, heart, thymus, spleen, kidneys, and adrenal glands, were below average for gestational age. Total body weight was 800 g (average = 1,096 g for 28 weeks; 95% range = 684-1,508 g), and crown-rump length was 18 cm (average = 25.7 cm; 95% range = 22.5-28.9 cm).

**Figure 3:**
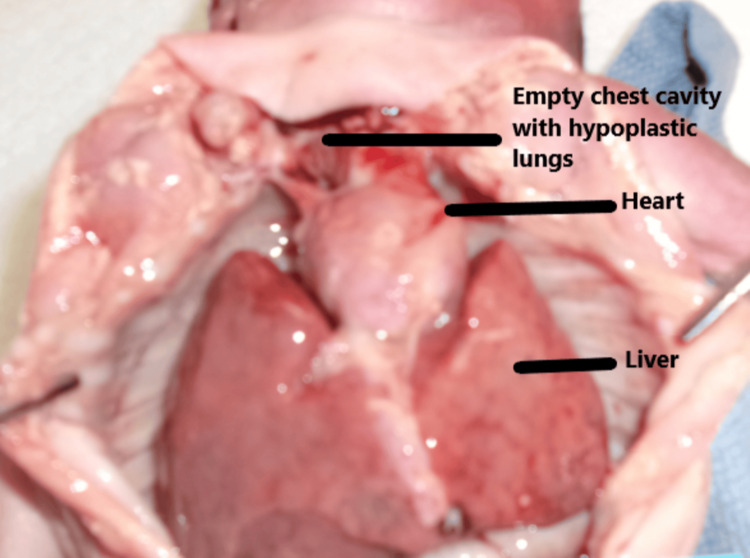
Bilateral hypoplastic lungs not visible in the chest cavity. The heart and liver are visible.

**Figure 4:**
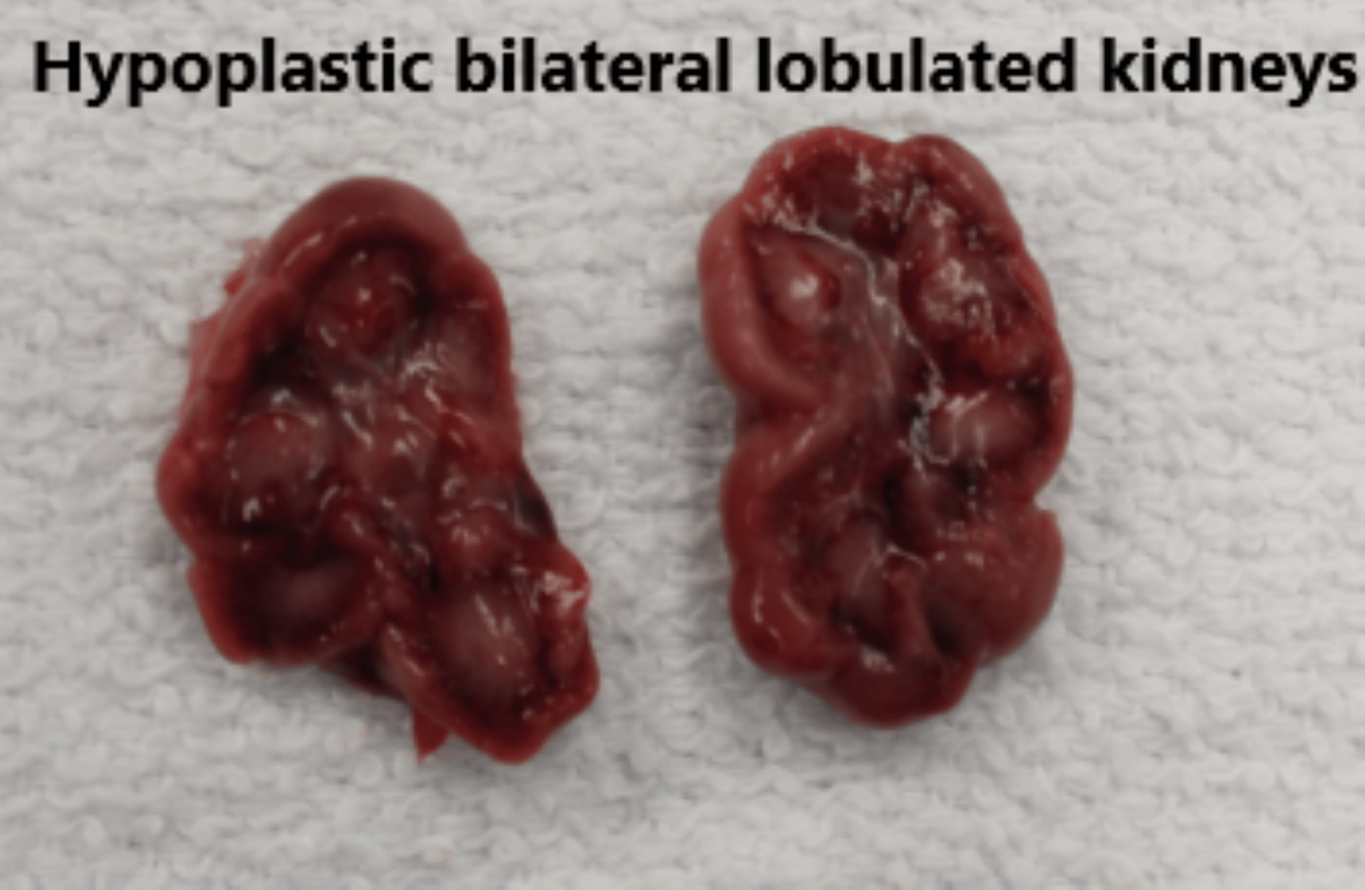
Bilateral lobulated kidneys.

**Figure 5:**
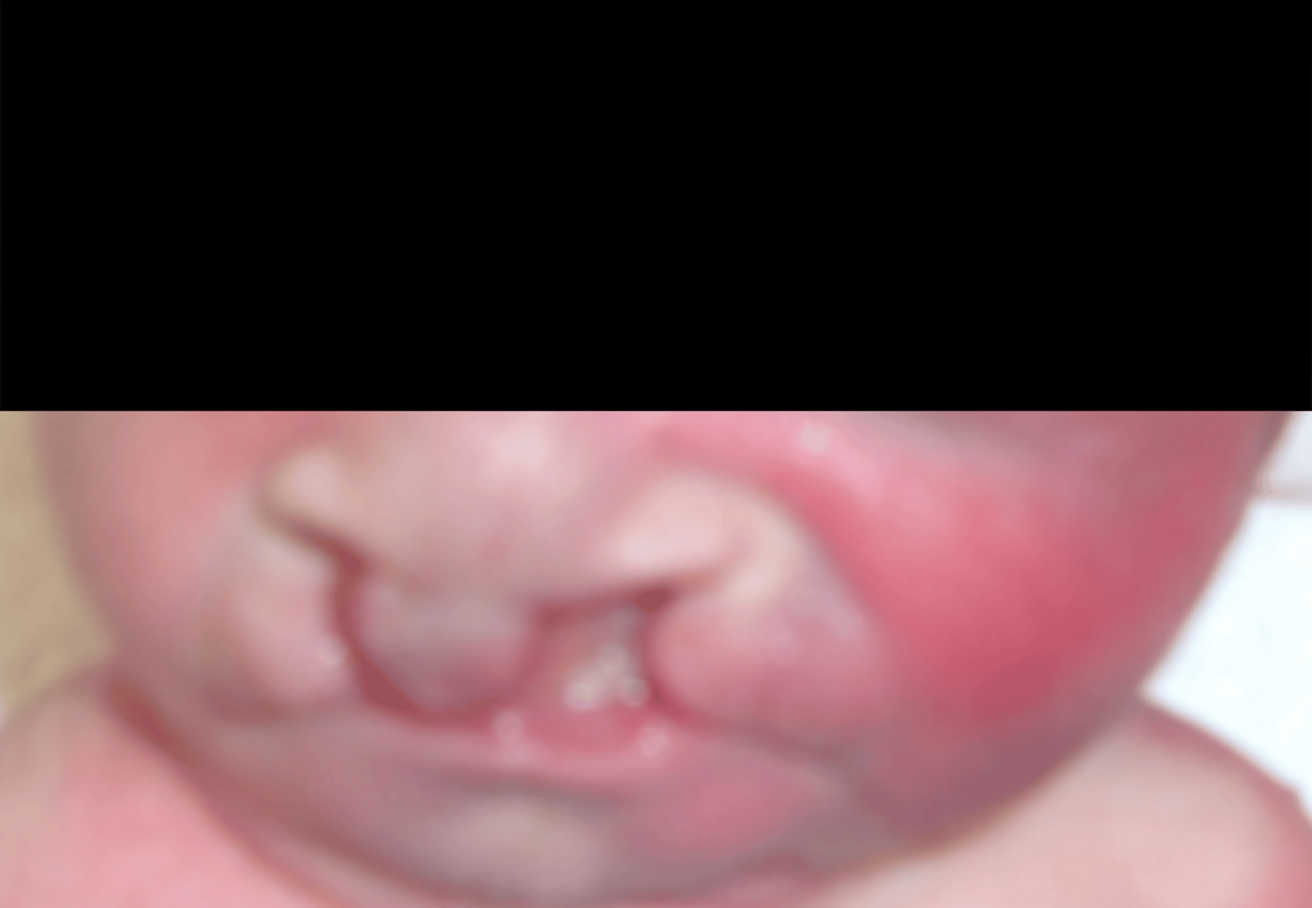
Bilateral cleft lip.

**Figure 6:**
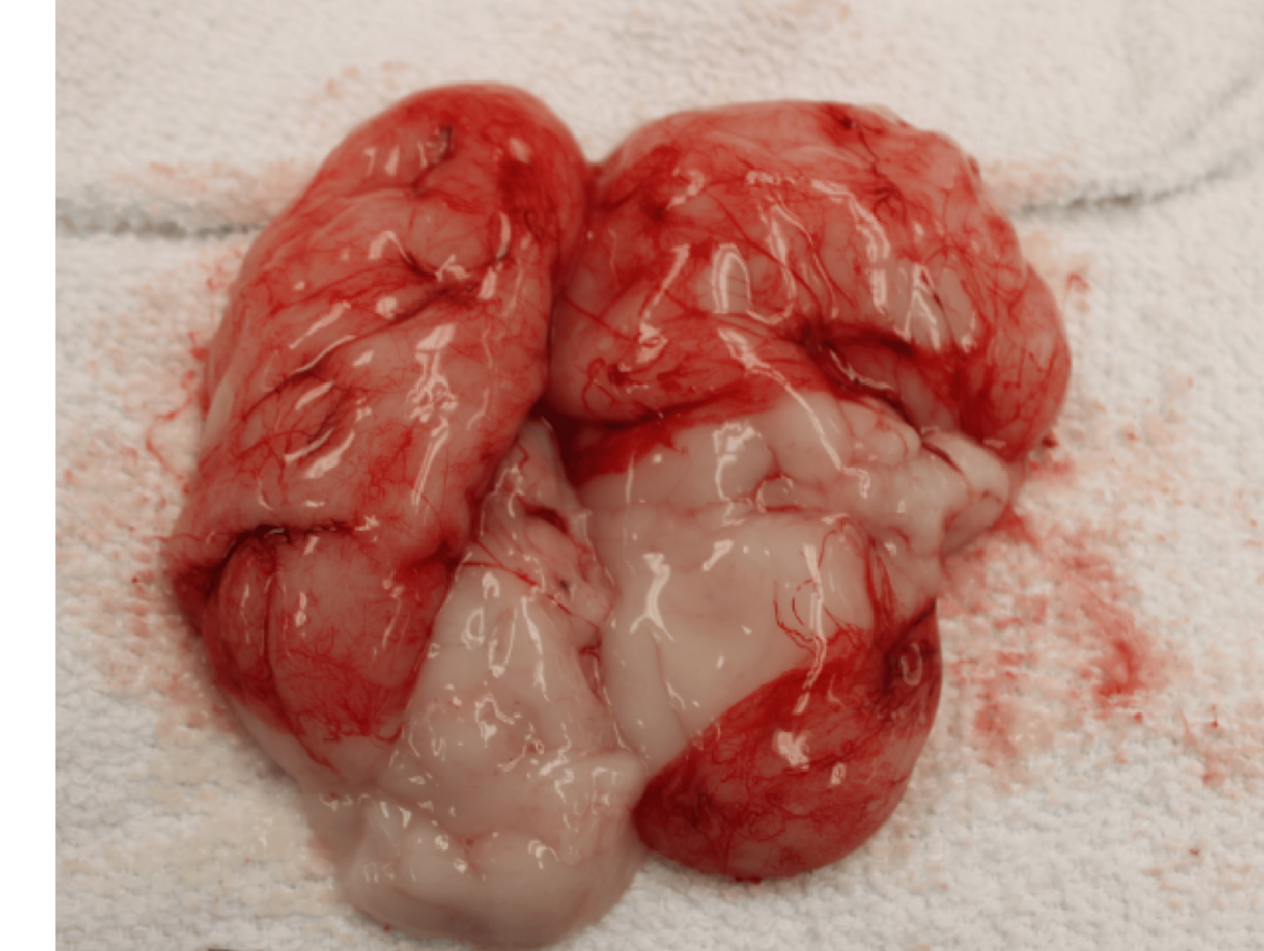
Brain with cerebellar hypoplasia and mixed microgyria-pachygyria pattern.

**Figure 7:**
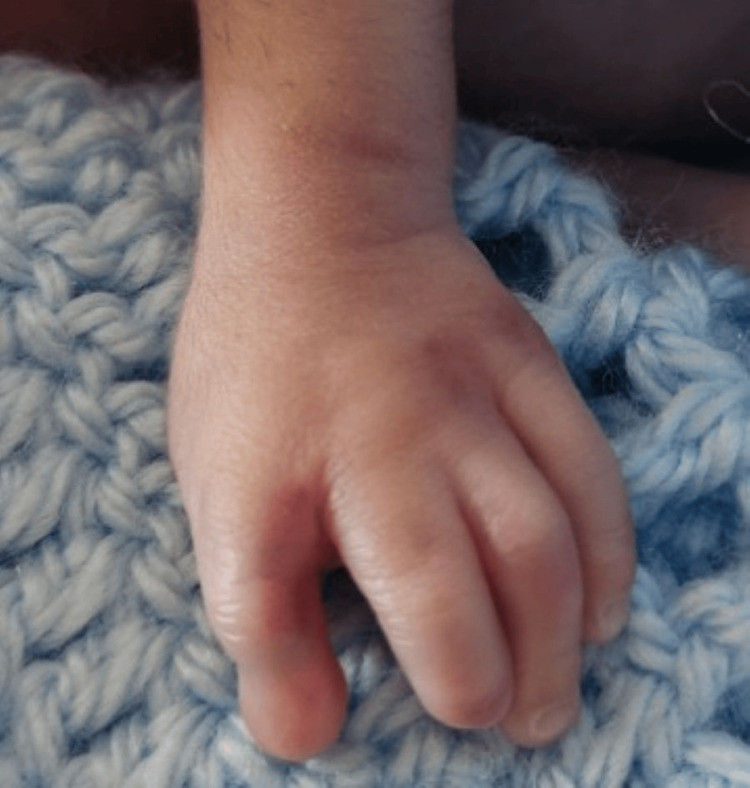
Hypoplasia of the fingernails and anomaly of the fingers.

Major autopsy findings included: (1) severe bilateral pulmonary hypoplasia, with lung weight of 1.11 g (reference = 10-44.8 g), although histologic maturation was appropriate for gestational age (Figure 3). (2) Biventricular hypertrophy. (3) Perimembranous VSD with mitral valve hypoplasia. (4) Complete bilateral diaphragmatic agenesis, likely the primary contributor to neonatal demise. (5) Bilateral lobulated kidneys with islands of adipose tissue (Figure 4). (6) Mild-to-moderate pleural and peritoneal effusions, ascites, and hepatic congestion. (7) Bilateral superior vena cavae. (8) Bilateral cleft lip and palate (Figure 5). (9) Brain weight below average with cerebellar hypoplasia and mixed microgyria/pachygyria pattern (Figure 6). (10) Hypoplastic fingernails and toenails (Figure 7). (11) Multiple external anomalies documented in gross photographs.

The family continued to be involved in post-natal counselling. At her postpartum follow-up visit, the mother was coping appropriately and expressed interest in pursuing fostering/adoption.

## Discussion

This case highlights not only the severe phenotype associated with the *RLIM* Arg611Cys mutation but also the importance of a coordinated multidisciplinary approach in supporting families facing a diagnosis of TOKAS. Given the rarity of this disorder, with fewer than 50 genetically confirmed cases described, the body of evidence guiding counseling and perinatal management remains limited [4,5,8]. As demonstrated in this case, individualized care integrating maternal-fetal medicine, fetal cardiology, neonatology, genetics, pathology, and perinatal palliative care is essential to ensure comprehensive assessment, anticipatory guidance, and family-centered support. Shared decision-making was central to this process, allowing the family to align the birth plan with their priorities, while early involvement of palliative care facilitated clear communication, memory-making, and psychosocial support.

Among known pathogenic *RLIM* variants, Arg611Cys is the most frequently reported and is consistently associated with a severe prenatal phenotype characterized by congenital anomalies, hydrops, and early lethality [2,4,6,9]. Cuinat et al. [4] described the largest fetal cohort of TOKAS to date and noted recurring findings such as facial dysmorphism, palate abnormalities, hypoplastic nails, congenital heart disease, thoracic anomalies, hydrops, and profound fetal growth restriction. Many of these manifestations were present in our case.

Certain findings in this infant, such as bilateral cleft lip and palate, lobulated kidneys, and bilateral superior vena cavae, have been reported infrequently or not at all in prior cohorts, further expanding the clinical spectrum of TOKAS. Notably, diaphragmatic agenesis and severe pulmonary hypoplasia were striking findings at autopsy and likely the leading contributors to early neonatal demise. Such anomalies are consistent with reports demonstrating the critical role of *RLIM*/*RNF12* in embryonic diaphragm, lung, and cardiac development [1,3,7]. Overall, the constellation of anomalies observed in this case aligns with the severe end of the phenotypic spectrum associated with loss-of-function or destabilizing *RLIM* variants, especially Arg611Cys.

Of the 18 antenatally diagnosed cases previously published, 12 involved the Arg611Cys substitution; with the addition of this case, 13 of 19 reported antenatal cases are now attributed to this single variant. Consistent with prior reports, all cases have been inherited from an asymptomatic carrier mother [4].

TOKAS is caused by pathogenic variants in the X-linked *RLIM* (*RNF12*) gene, which encodes an E3 ubiquitin ligase involved in transcriptional regulation, stem cell differentiation, and X-chromosome inactivation. Functional studies demonstrate that *RLIM* is dosage-sensitive and plays a critical role during early embryogenesis, particularly in the development of the brain, diaphragm, heart, and musculoskeletal system [1,3,9].

Variant Arg611Cys lies within a highly conserved region essential for ubiquitin ligase activity and protein stability. Experimental studies show that variants affecting this region, including Arg611Cys, result in impaired protein function, dysregulated developmental signaling, and widespread disruption of organogenesis [1,6,9]. These molecular findings correlate with the multisystem anomalies and prenatal lethality frequently observed in affected male fetuses.

Once a maternally inherited *RLIM* pathogenic variant is identified, recurrence risk counseling becomes essential. As an X-linked disorder, each future pregnancy has a 50% chance of inheriting the mutation, with male offspring having a 50% chance of being affected and female offspring having a 50% chance of being carriers [4,5,8,9].

Female carriers are generally asymptomatic but may have subtle or variable findings such as short stature, mild skeletal differences, or menstrual or ovarian dysfunction [4]. Given the recurrence risk and severity of outcomes in affected males, options such as preimplantation genetic testing, in vitro fertilization, early invasive prenatal diagnosis, or targeted cfDNA screening may be considered in future pregnancies.

Because of the nature and severity of TOKAS, many affected pregnancies likely go undiagnosed. Severe cases may result in miscarriage, intrauterine fetal demise, stillbirth, or death shortly after birth, and a definitive diagnosis cannot be established without WES [1,3,4]. Limited access to advanced prenatal imaging, specialized genetic testing, or postmortem evaluation, as well as personal preference to decline testing, further contributes to the under-recognition of this condition.

As TOKAS is uniformly associated with poor perinatal survival when severe anomalies or hydrops are present, early involvement of a multidisciplinary team is crucial. In this case, maternal-fetal medicine guided antenatal imaging and diagnostic testing, genetics facilitated interpretation of the *RLIM* variant and recurrence counseling, neonatology provided anticipatory guidance regarding expected postnatal instability, and palliative care ensured that the family’s goals and values shaped the overall plan. Memory-making activities, psychosocial support, and bereavement resources were essential components of holistic care.

This case underscores how coordinated, compassionate, and comprehensive care can honor family goals while navigating the clinical uncertainty and emotional complexity of a rare, life-limiting diagnosis.

## Conclusions

TOKAS is an underrecognized rare genetic disorder and is uniformly associated with poor perinatal survival. In this case, obtaining a diagnosis provided clarity regarding prognosis and allowed the care team to guide decision-making in alignment with the family’s goals. When combined with serial ultrasound findings, the genetic diagnosis supported individualized, compassionate, and realistic counseling. This approach required a coordinated multidisciplinary effort across maternal-fetal medicine, genetics, neonatology, pathology, and palliative care. Our experience underscores the importance of comprehensive diagnostic evaluation and integrated team-based care when managing pregnancies complicated by rare, life-limiting genetic conditions.
